# The Therapeutic Potential of Tackling Tumor-Induced Dendritic Cell Dysfunction in Colorectal Cancer

**DOI:** 10.3389/fimmu.2021.724883

**Published:** 2021-10-06

**Authors:** Beatriz Subtil, Alessandra Cambi, Daniele V. F. Tauriello, I. Jolanda M. de Vries

**Affiliations:** ^1^ Department of Cell Biology, Radboud Institute for Molecular Life Sciences, Radboud University Medical Center, Nijmegen, Netherlands; ^2^ Department of Tumor Immunology, Radboud Institute for Molecular Life Sciences, Radboud University Medical Center, Nijmegen, Netherlands

**Keywords:** metastatic colorectal cancer, cancer immunity, dendritic cell defects, immunotherapy, tumor microenvironment, immunosuppression, patient-derived organoids

## Abstract

Colorectal cancer (CRC) is the third most diagnosed malignancy and the second leading cause of cancer-related deaths worldwide. Locally advanced and metastatic disease exhibit resistance to therapy and are prone to recurrence. Despite significant advances in standard of care and targeted (immuno)therapies, the treatment effects in metastatic CRC patients have been modest. Untreatable cancer metastasis accounts for poor prognosis and most CRC deaths. The generation of a strong immunosuppressive tumor microenvironment (TME) by CRC constitutes a major hurdle for tumor clearance by the immune system. Dendritic cells (DCs), often impaired in the TME, play a critical role in the initiation and amplification of anti-tumor immune responses. Evidence suggests that tumor-mediated DC dysfunction is decisive for tumor growth and metastasis initiation, as well as for the success of immunotherapies. Unravelling and understanding the complex crosstalk between CRC and DCs holds promise for identifying key mechanisms involved in tumor progression and spread that can be exploited for therapy. The main goal of this review is to provide an overview of the current knowledge on the impact of CRC-driven immunosuppression on DCs phenotype and functionality, and its significance for disease progression, patient prognosis, and treatment response. Moreover, present knowledge gaps will be highlighted as promising opportunities to further understand and therapeutically target DC dysfunction in CRC. Given the complexity and heterogeneity of CRC, future research will benefit from the use of patient-derived material and the development of *in vitro* organoid-based co-culture systems to model and study DCs within the CRC TME.

## Introduction

Colorectal cancer (CRC) is one of the most common and deadliest cancers worldwide (1, 2). At early stages of localized disease, surgical resection leads to a good prognosis or even cure (3). Unfortunately, more than half of the CRC patients develop metastasis, either at the time of diagnosis or later as relapse (4). The most common metastatic site is the liver, but metastasis can also be found in the lungs, peritoneum, bones, and brain. Distant metastases are increasingly resected with curative intent (5, 6). For unresectable or recurrent metastatic disease, standard chemotherapies, as well as targeted treatments, have improved median overall survival to over 30 months (7–9). Despite these significant advances, metastatic CRC (mCRC) patients remain largely unresponsive to (immuno)therapy resulting in a 5-year survival rate of only 12% (1). At the moment, different therapeutic approaches are being actively investigated to fulfil the unmet need for therapies in mCRC (10, 11).

As with most cancers, CRC develops and spreads by evading immunosurveillance. To subvert the immune system, tumors evolved a number of escape mechanisms including loss of tumor antigens, upregulation of inhibitory molecules, and the generation of an immunosuppressive environment which recruits and corrupts stromal and immune cells (12). The CRC tumor microenvironment (TME) prevents immunosurveillance, supporting tumor growth and progression (13, 14). Besides hampering anti-tumor immunity, the generation of an immunosuppressive environment also hinders the success of immunotherapies (10, 14).

Dendritic cells (DCs), also known as professional antigen presenting cells, are a key immune cell type often impaired by the immunosuppressive TME. DCs are the central players in triggering, coordinating and amplifying anti-tumor immune responses, and in driving the clinical success of immunotherapies (15–17). However, in the presence of immunosuppressive signals, such as the ones released by tumor cells, DCs become dysfunctional and induce tolerance. Several studies suggest that tumor-mediated impairment of DC functions is decisive for immune evasion, tumor growth, metastasis initiation, and treatment resistance in different cancers including CRC (17–23). Despite the key role of functional DCs in anti-tumor immunity and treatment response, it is still largely unclear how CRC shapes DC fate.

The main aim of this review is to provide an overview of the current knowledge on how primary and metastatic CRC-driven immunosuppression affects DC phenotype and functionality. We will assess how this correlates with disease progression, mCRC patients’ prognosis and treatment response. Moreover, the potential of therapies to revert DC defects in CRC will be discussed. From there, knowledge gaps will be pinpointed as unexplored avenues to study and target DC dysfunction in CRC.

## Colorectal Cancer

CRC comprises a highly complex, heterogenous, and lethal group of diseases. Several factors contribute to CRC development and have implications in treatment response. As mentioned, the survival and treatment options of CRC patients largely depend on the stage, i.e., the extent of tumor invasion and spread at the time of diagnosis. Outgrowth of metastasis is facilitated by synchronous undetectable disseminated metastatic cells, tumor shedding into circulation, and therapy-induced immune impairment. As such, early detection of CRC through screening becomes a crucial factor to reduce mortality of the disease. However, disease stage is not always predictive for patient response and outcome. CRC patients at the same stage might have different disease progression based on the molecular heterogeneity of the tumor and the composition of the TME (24–27).

### Tumorigenesis

CRC develops from a multistep accumulation of genetic and epigenetic alterations (28). The majority of CRC cases arise from sporadic mutations due to an interplay of environmental and lifestyle factors, the remaining due to genetic predisposition (29, 30). CRC develops from abnormal proliferation of mucosal epithelial cells of the large intestine, named polyps or adenomas, which can evolve to adenocarcinomas. CRC can develop from adenoma to carcinoma through one or a combination of different molecular mechanisms, namely chromosomal instability, CpG island methylation, and DNA mismatch-repair deficiency (24). Different sequential driver mutations associated with tumorigenesis occur in the APC/β-catenin, KRAS, MAPK and BMP/TGF-β pathways, as well as in tumor suppressor genes, such as TP53, at later stages (31, 32). As mutations accumulate, adenocarcinomas become invasive and spread to distant sites in the body establishing metastasis.

Depending on the underlying driving mechanism of genomic instability, CRC tumors can be broadly classified into (1) microsatellite instability-high (MSI-H), which account for ~15% of tumors and (2) microsatellite stable (MSS) accounting for the remaining ~85% of the cases (33). MSI tumors are characterized by high frequency of replication errors due to defective DNA mismatch repair mechanisms, which lead to a hypermutated state. Typically, MSI tumors are highly immunogenic, present high percentage of immune infiltrates, and are associated with a more favorable prognosis (34). In contrast, MSS tumors, which account for the large majority of CRC cases, are poorly immunogenic with low mutational burden, and are linked to poor prognosis (31, 32).

In mCRC, treatment with immune checkpoint inhibitors presents promising responses only in a minority of patients, with MSI tumors, while MSS tumors do not respond to PD-1 or PD-L1 inhibitors (35, 36). Unresponsiveness of MSS tumors to immune checkpoint inhibition and other immunotherapies has been associated with the low number of tumor-specific neoantigens, lack of infiltrating immune cells, and tumor-mediated immunosuppression (37). This implies that, to date, the vast majority of mCRC patients does not qualify for immunotherapy.

Besides MSS/MSI stratification, a more comprehensive classification system for CRC has been developed. The Consensus Molecular Subtype system, which divides CRC patients in 4 subtypes based on transcriptome analysis of the tumor and associated stromal and immune cells (38, 39). This stratification system suggests that characterizing gene expression of not only tumor cells but also of surrounding tumor-associated cells (such as fibroblasts, leukocytes and endothelial cells) allows better stratification of patients and confers higher predictive value for prognosis, management, and selection of appropriate treatment (40). This emphasizes the importance of studying both the tumor and its surroundings.

### The Tumor (Immune) Microenvironment

CRC initiation, progression, metastatic dissemination, and treatment resistance is not only driven by the accumulation of genomic and epigenomic aberrations but also by intricate and dynamic interactions between malignant and neighboring cells in the TME (41–44). Surrounding cells comprise endothelial cells, gut microbiota, cancer-associated fibroblasts (CAFs), and immune cells including tumor-associated macrophages (TAMs), myeloid-derived suppressor cells (MDSCs), natural killer (NK) cells, DCs, and T cells (45). Tumor cells are well-known for having a strong modulatory effect, for being able to recruit, corrupt or re-educate surrounding cells towards tumor-promoting phenotypes that foster tumor growth and spread.

Importantly, CRC generates a strong immunosuppressive TME that hampers immunosurveillance and allows immune evasion. To escape eradication by the immune system CRC recruits and polarizes immunosuppressive regulatory T cells (Tregs), TAMs, and MDSCs. In addition, CRC inhibits or excludes immune cells with anti-tumor potential such as DCs, NK cells, and effector T cells from the TME. By regulating local and systemic immune function, CRC creates immune impairments and an environment propitious for tumor growth and dissemination (46). Several studies have reported that CRC-induced local and systemic immune dysfunctions are closely associated with patient prognosis and sensitivity to therapy (47–51).

The intricate web of interactions within the TME is mediated by cell-to-cell contact and soluble factors, such as cytokines, chemokines, and growth factors, derived from the tumor and activated surrounding cells. These factors are constantly remodeling the TME and have not only local but also systemic effects, which are crucial for generalized immunosuppression enabling the generation of pre-metastatic niches and successful metastatic establishment (52).

One key immunosuppressive signaling molecule associated with CRC is transforming growth factor-β (TGF-β). High TGF-β expression in the TME of CRC has been linked to poor prognosis with a crucial role in successful tumor progression and metastasis development (40, 53–59). TGF-β modifies the TME by regulating infiltration and by suppressing or tweaking the phenotype of immune cells towards tolerance, impeding anti-tumor immunity (53, 60, 61). Consistent with these findings, neutralization of TGF-β signaling in the TME was found to impair liver metastasis establishment by unleashing T cell and NK cells anti-tumor responses, in different pre-clinical CRC models (14, 40, 54, 55, 58, 62). Promisingly, in a metastatic mouse model for CRC, treatment of established metastasis with anti-PD-L1 antibodies in combination with TGF-β blockade resulted in potent curative anti-tumor T cell-mediated immune responses (14). Besides TGF-β, other factors such as IL-6, IL-33, IL-8, IL-23, PGE2 and IDO-1 have shown similar immunomodulatory properties and impact in metastasis development and patient prognosis in CRC (63–74). These studies highlight the role and the potential of further exploring the interactions between malignant cells and immune cells, and combining current therapies with agents designed to target the TME (10, 13).

It is clear that regulation of immune cells by the immunosuppressive TME generated by CRC has a pivotal role in disease progression, not only by hampering immuno-surveillance but also by compromising the effectiveness of immunotherapies. Since, no effective treatment is available for the majority of mCRC patients, it becomes imperative to further understand how CRC interferes with immune activation for identification of new mechanisms for complementary therapies. DCs, as the main orchestrators of innate and adaptive anti-cancer immunity, appear as promising targets to unleash immune responses and immunotherapy efficacy.

## Dendritic Cells: The Hub of Anti-Tumor Immunity

DCs comprise a heterogenous population of cells specialized in antigen capture, processing, and presentation. DCs act as hub of the immune system by initiating, linking and coordinating innate and adaptive immune responses (15, 16). DCs are key regulators of specific immune response owing to their unique capacity to (cross-)present antigens and prime T cells (15, 20). In anti-tumor immunity, DCs can promote T cell and NK cytotoxic activities, and also exert direct tumoricidal activity, sustaining cancer immunosurveillance. Consequently, DCs have been shown to have a crucial role in inhibiting local tumor growth, tumor dissemination, and metastatic establishment (17).

Tissue-resident DCs in steady-state conditions scan the environment for antigens and danger signals, acting as sentinels. In homeostatic conditions or under suppressive environmental cues, DCs present an immature and tolerogenic phenotype, characterized by low expression of co-stimulatory molecules and pro-inflammatory cytokines, inability to prime T cells, and secretion of immunosuppressive cytokines (e.g., IL-10 and TGFβ). This functional state ensures immune (self-)tolerance, through various mechanisms including T cell depletion and anergy, as well as generation of Tregs (15, 75–77).

If uptake and processing of (tumor) antigens occurs in the presence of danger signals and inflammatory cytokines, DCs undergo maturation (78). The maturation process encompasses several morphological, functional, and phenotypical changes, which include enhanced migration abilities through CCR7 expression, upregulation of co-stimulatory molecules CD80, CD83, and CD86, and secretion of pro-inflammatory cytokines such as IL-12, IL-6, TNF-α and IL1-β. All these signals, together with antigen presentation on major histocompatibility complexes (MHC), are required for proper priming, activation, and proliferation of T cells, and induction of an antigen-specific response (15, 75, 76). Upon maturation, DCs migrate to a lymph node, where they prime and activate antigen/tumor-specific T cells. Subsequently, T helper (Th) or cytotoxic T cells (CTL), migrate into the tumor site where they can perform their effector functions (15, 79).

Additionally, tumor-infiltrating DCs (TIDCs) have been reported to regulate the magnitude and duration of T cell responses within the TME, either through direct antigen presentation or establishment of a favorable cytokine environment, in different tumor models including breast cancer and melanoma (15, 20, 79–84). Antigen presentation within tumors might occur in ectopic tertiary lymphoid structures (TLS), which are hypothesized to play an important role in response to neoantigens that form during later stages of tumor progression (15). It seems that in the cancer setting, tumor-draining lymph node DCs might initially prime naïve T cells, while later intra-tumoral DCs further license and activate T cells in the tumor bed (85). Overall, these studies indicate that TIDCs are required for recruitment, re-priming, and re-stimulation of T cells to acquire full effector function in the TME (20).

DCs have an additional role in innate anti-tumor immunity, through modulation and enhancement of NK cell activity. On the one hand, mature DCs potentiate NK cytotoxicity against tumor cells by secreting pro-inflammatory cytokines (e.g., IL-12, IL15) and cell-to-cell contact. On the other hand, NK cells promote DC infiltration into tumors, as well as their maturation, cytokine-producing ability, migratory potential, and facilitate cross-presentation through the secretion of chemokines and growth factors such as CCL5, XCL1, and FLT3L. This interaction in turn results in enhanced and stronger anti-tumor T cell activation. The dynamic crosstalk between DC and NK takes place in both tumor-draining lymph nodes and in the TME (74, 86, 87). In fact, an optimal anti-tumor immune response appears to rely on effector T cells and NK cells, which are jointly induced and coordinated by DCs (86). DCs are thus essential mediators for the induction of powerful immune responses against cancer cells (88).

Notably, DCs encompass a highly complex and heterogenous population. Regarding their origin and differentiation pathway, four major lineages can be defined: myeloid or conventional DCs (cDC1 and cDC2), plasmacytoid DCs (pDC), inflammatory or monocyte-derived DCs (MoDC), and Langerhans cells (LC). Even though all DCs harbor antigen-presenting and T cell activating abilities, the different subsets present distinct phenotypes and specialized functions. This expands the range and flexibility of immune responses (21, 73, 89). For instance, cDC1 are specialized in cross-presentation and CD8+ T cell responses, cDC2 in CD4+ T cell priming, pDCs in type I interferon-mediated responses, and MoDCs perform different functions in inflammatory settings (15, 21, 89–93).

As such, the immunogenic or tolerogenic functions of DCs, T cell priming or tolerance, depend on their functional subset and their maturation status, which is dictated by environmental cues (75, 77, 79, 88, 94). The phenotypic and functional plasticity of DCs renders them susceptible targets for the evolution of tumor-mediated suppressive mechanisms (95).

## Tumor-Induced DC Dysfunction

DCs can play either a regulatory, tolerogenic function or coordinate potent immune responses, depending on the local tumor milieu. Tumors take advantage of this functional plasticity by interfering with DC functions and shifting the balance towards immune evasion (22). As such, tumors employ a variety of mechanisms to disrupt DC functions, mainly mediated through the immunosuppressive TME, that compromise the development of anti-tumor immune responses and facilitate local and metastatic progression (79).

**Figure 1:**
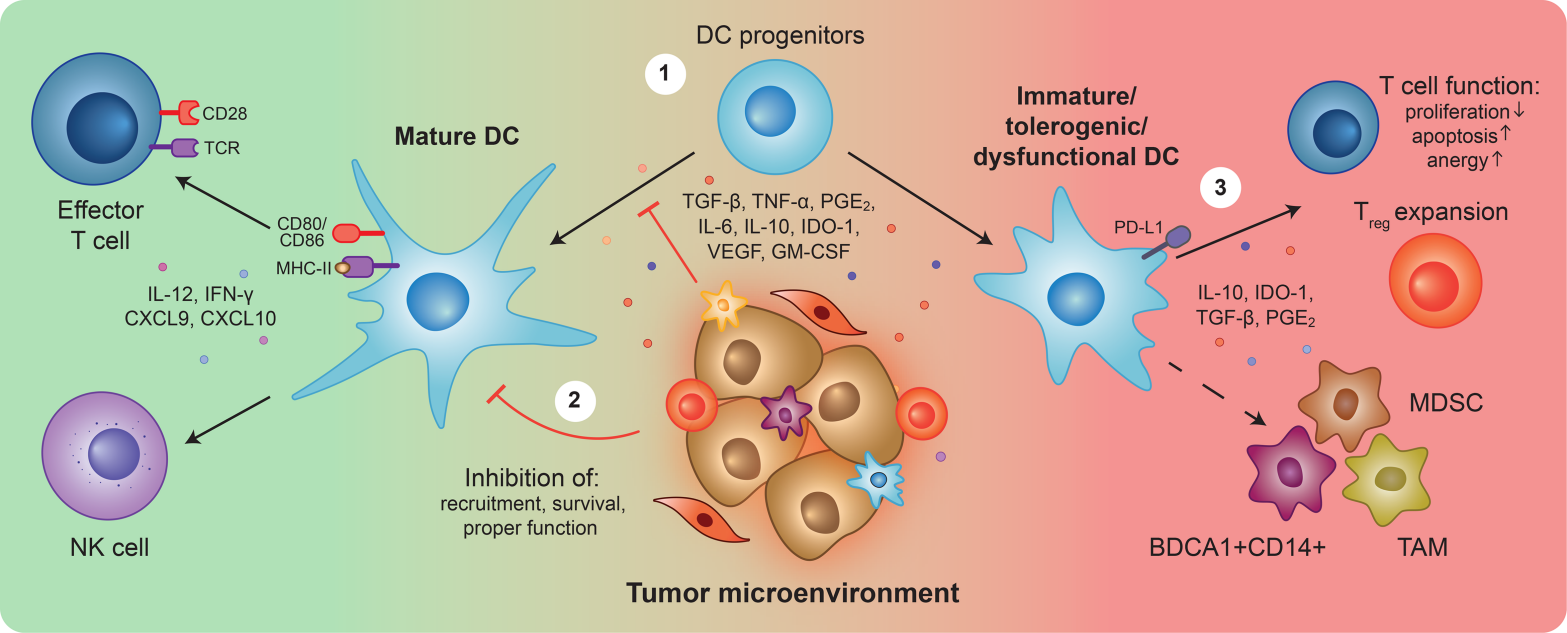
Overview of Dendritic cell (dys)functions in cancer. Upon detection of tumor antigens and danger signals, dendritic cells (DCs) become activated, upregulate co-stimulatory surface molecules and secrete pro-inflammatory cytokines. Mature DCs can (cross)-present antigens, trigger tumor-specific T cell responses, and stimulate natural killer (NK) cell activity to unleash cytotoxic anti-tumor immunity (left). During tumor development and progression, the release of tumor-derived suppressive factors prevents DC progenitors from properly differentiating ①, and differentiated DCs from fulfilling their functions ②. Resulting immature, tolerogenic and/or dysfunctional DCs, characterized by the expression of TGF-β, IL-10, IDO-1, PGE2, and PD-L1, can inhibit T cell anti-tumor responses ③. Furthermore, they can differentiate into and favor the expansion of immunosuppressive populations such as myeloid-derived suppressor cells (MDSCs), BDCA1+CD14+ cells, and tumor-associated macrophages (TAMs). Overall, the impairment of DCs is a crucial step for tumor immune evasion, triggering a cascade of immunosuppression that hampers anti-tumor immunity and creates a propitious environment for tumor growth and metastasis initiation.

Quantitative and functional impairments of circulating and intra-tumoral DCs have been widely observed in several types of malignancies including melanoma, breast, pancreatic, ovarian, colorectal, prostate, and lung cancer (16). Several studies, have elucidated on escape mechanisms employed by tumors to disrupt DC functions at different levels (**Figure 1**):

1) Impairing proper differentiation of DCs from hematopoietic and myeloid precursors leading to decreased local and circulating DC numbers. Instead, tumors favor differentiation of precursors into immunosuppressive populations, such as MDSCs, TAMs, and BDCA1+CD14+ cells, which further contribute to an immunosuppressive environment (23, 73, 96–104). 2) Inducing apoptosis or favoring exclusion of mature DCs from the TME, for instance, by blocking NK-mediated recruitment of DCs (74, 105). Furthermore, inhibiting proper DC maturation and activation, preventing expression of co-stimulatory molecules, and secretion of pro-inflammatory cytokines, blocking DCs at an immature state. Several tumor-derived factors have been shown to mediate DC defects including TGF-β, TNF-α, IDO-1, PGE2, IL-6, IL-10, VEGF, and GM-CSF (70, 106–116). These factors hinder DC migration, antigen presentation, and effective T cell and NK activation (105–108, 113, 116–128). 3) Directly and indirectly inhibiting anti-tumor T cell functions. The TME skews DCs from immunostimulatory into tolerogenic and immunosuppressive phenotypes and functions, affecting T cell survival and proliferation, or by inducing anergy (an hyporesponsive state) and the expansion of Treg cells. These processes are characterized by the increased expression of immunosuppressive factors such as TGF-β, IL-10, PGE2, IDO-1, and PD-L1 by DCs (104, 114, 115, 129–142).

Overall, the presence of an immunosuppressive TME induces DC-mediated tolerance rather than immunity, contributing to immune escape and dampening of anti-tumor T cell responses. In different studies, these effects on DCs have translated into accelerated tumor progression, increased tumor-draining lymph node metastasis, immunotherapy failure, systemic dysfunctional immune status, and poor prognosis (102, 116, 120, 143–146). In summary, evidence suggests that tumor-induced DC defects are decisive for tumor growth, metastasis initiation, and prognosis. This emphasizes the impact of a phenotype shift in DCs in driving either immunosurveillance or accelerated malignant growth.

## Numerical and Functional Defects of DCs in CRC Patients

Given the existence of different DC subsets and functional states, their plasticity in regard to signals from the TME and the consequent impact on anti-tumor immunity, it is not surprising that DC phenotypical changes and defects have shown clinical relevance across different tumor types (22, 147, 148). DCs have been widely investigated in CRC patients with variations in number, phenotype, and function of both circulating and TIDCs reported (**Table 1**).

**Table 1:** Overview of studies investigating tumor-infiltrating (TIDCs) and circulating dendritic cells (DCs) in colorectal cancer (CRC) patients.

CRC (n)	Experimental setup	DC characterization	Key conclusions	Reference
***TIDCs – interactions with other immune cells and correlations with disease progression and prognosis***				
121	Tissue IHC	S100	↑ S100+ DCs ↔ good prognosis, higher survival, often without metastasis and ↑ lymphocyte infiltration	(Ambe, Mori, & Enjoji, 1989) (149)
30	Tissue IHC	S100	↑ S100+ DCs ↔ good prognosis ↓ S100 +DCs ↔ lymph node and hepatic metastasis, >stage III	(Nakayama et al., 2003) (150)
104	Tissue IHC	S100 and HLA-II	↑ S100+ DCs ↔ ↑ T cell infiltration and disease-free survival	(Dadabayev et al., 2004) (151)
40	Tissue IHC	S100, CD11c, CD208, CD209, CD123, and CD1a	S100+ DCs ↔ Tregs ↑ S100+ DCs ↔ prolonged survival ↓ S100+ DCs ↔ worse prognosis	(Nagorsen et al., 2007) (152)
16	Tissue IHC	CD205	↓ CD205+ DCs and high HMGB1 expression by CRC ↔ lymph node metastasis	(Kusume et al., 2009) (153)
52	Tissue IHC	CD11c+	↓ CD11c+ myeloid DCs ↑ Tregs ↔ tumor invasion, advanced stage, lymph node metastasis and poor prognosis	(Gai, Li, Song, Lei, & Yang, 2013) (154)
63	Tissue IHC	CD123 (pDCs)	↑ pDC/myeloid DC ratio and ↑ Tregs ↔ lymph node metastasis	(Gai, Song, Li, Lei, & Yang, 2013) (155)
149	Tissue IHC	BDCA-2+ (pDCs)	↑ pDC ↔ TLS and prolonged survival	(Kießler et al., 2021) (156)
58	Flow cytometry and RNA sequencing	BDCA-2+ (pDCs)	↑ pDC ↓innate lymphoid cells ↔ advanced disease stage	(Wu et al., 2021) (157)
***TIDCs – maturation status and distribution***				
57	Tissue IHC	CD83, HLA-DR, CD40, and CD86	Density of mDCs: Normal mucosa > primary CRC > metastatic CRC No association with TGF-β or IL-10	(Schwaab, Weiss, Schned, & Barth, 2001) (158)
17	Tissue IHC	CD83 and CD1a	CD83+ mDCs: present in the invasive margin and cluster with T cells CD1a+ iDCs: scattered in the tumor stroma	(Suzuki et al., 2002) (159)
60	Tissue IHC	CD1a, S100, CD83, and HLA-DR	CD83+ mDCs: present around metastases and in the sinusoidal lumen CD1a+ iDCs: scattered in the tumor stroma	(M. Gulubova, Manolova, Cirovski, & Sivrev, 2008) (160)
26	Tissue IHC and gene expression	CD83	Primary site and lymph nodes: ↓ CD83+ mDCs ↔ high COX2 and IL-6	(Cui et al., 2007) (161)
23	Tissue IHC and gene expression	CD1a, CD83, and CD208	↓ CD83+ CD208+ mDCs ↑ CD1a+ iDCs ↔ increasing COX2 expression	(Yuan et al., 2008) (162)
69	Tissue IHC	S100, CD208	In MSI tumors in comparison with MSS: ↑ CD208+ mDCs and ↓ Tregs	(Bauer et al., 2011) (163)
133	Tissue Gene expression	Genes implicated in immune response	In MSI tumors in comparison with MSS: ↑ co-stimulatory molecules in DCs	(Banerjea et al., 2004) (164)
***TIDCs – maturation status and correlations with disease progression and prognosis***				
70	Tissue IHC	CD83	↓ CD83+ mDCs ↔ poor prognosis	(Miyagawa et al., 2004) (165)
22	Tissue IHC	CD83	↓ CD83+ mDCs ↔ advanced disease and lymph node metastasis ↑ CD83+ mDCs and IL-12 expression ↔ better prognosis	(Inoeu et al., 2005) (166)
142	Tissue IHC	HLA-DR, CD1a, and CD83	↓ CD83+ mDCs ↔ shorter survival ↔ TGF-β expression by CRC	(Maya Gulubova et al., 2010) (56)
86	Tissue IHC	HLA-DR, CD1a, and CD83	Metastasis in comparison to metastasis-free samples: ↓ CD83+ mDCs and ↑TGF-β	(Maya Gulubova et al., 2013) (167)
44	Tissue IHC	CD1a and DC-LAMP	↓TILs ↑ CD1a+ iDCs/DC-LAMP+ mDCs ratio and KRAS mutation ↔ higher risk of disease recurrence	(Kocián et al., 2011) (168)
145	Tissue IHC	CD1a, S100, CD83, and HLA-DR	↓ CD83+ HLA-DR+ mDCs in invasive margin ↔ advanced stage (metastasis) and worse prognosis	(Maya V. Gulubova et al., 2012) (169)
556	Gene expression	Several DC-related genes	↑ mDCs ↑ T cells ↔ low risk group	(M. Li et al., 2020) (170)
473	Gene expression	CD80, CD83, and CD86	↑ CD80+, CD83+, CD86+ mDCs ↔ CXCL8 expression by CRC	(E. Li et al., 2021) (171)
326	Gene expression	Several DC-related genes	↑ DCs, IL-12 and in TLS ↔ strong Th1 and CTL response and more favorable prognostic	(Coppola et al., 2011) (19)
104	Tissue IHC	S100, CD1a, CD208, and HLA- II	↑CD208+ mDCs in the stroma ↔ shorter overall survival ↑CD1a+ iDCs in the advancing margin ↔ shorter disease-free survival	(Sandel et al., 2005) (172)
71	Tissue IHC	CD83	↑ mDCs ↔ tumor invasion and lymph node metastasis	(Pryczynicz et al., 2016) (173)
221	Tissue IHC	CD11c and PD-L1	↑ CD11c+ PD-L1+ DCs ↔ good survival and ↑ CD8+ T cell density	(Miller et al., 2021) (174)
***Blood circulating DCs - Numerical defects***				
106	Flow cytometry	HLA-DR and CD86	↓ Circulating DC ↔ ↑ TGF-β levels	(Huang et al., 2003) (175)
54	Flow cytometry	HLA-DR, CD11c, CD83, and CD86	Numerical and functional impairment of DC progenitors ↔ stage of the disease and ↑ VEGF levels	(Della Porta et al., 2005) (176)
27	Flow cytometry	BDCA-1, BDCA-2, BDCA-3, CD80, CD86, and HLA-DR	DCs number: healthy > metastatic > non-metastatic > chemotherapy treated subjects	(Bellik et al., 2006) (177)
26	Flow cytometry	CD33 and CD123	↓ CD123+ pDCs ↔ advanced stage	(Orsini et al., 2014) (178)
***Blood circulating DCs - Functional defects***				
31	Flow cytometry, functional assays	CD11c, CD123 HLA-DR, CD80, CD86, and CD83	↑ immature myeloid cell progenitors Defective DC maturation *ex vivo* ↔ ↑ VEGF Anti-VEGF antibody treatment: ↑ *ex vivo* stimulatory capacity of DC ↔ ↑ antigen-specific allogenic T cell proliferation	(Osada et al., 2008) (179)
23	Flow cytometry, functional assays	CD40, CD80, and CD83	Defective generation of mature and functional DC *ex vivo* ↔ advanced disease stage ↓ Ability to present antigens to allogeneic T cells ↑IL-10 ↓IL-12 and TNF-α	(Orsini et al., 2013) (180)
16	Flow cytometry, functional assays	CD83 CD1a HLA-DR CD86 FITC, CD80, CD209, and CD206	Defective DC maturation *ex vivo*	(Maciejewski et al., 2013) (181)
30	Flow cytometry, functional assays	CD80, CD11c, HLA-ABC, HLA-DR, CD14, CD133, CD11b, CD209, and CD86	Defective DC maturation *ex vivo* ↓IL-12	(Hsu et al., 2018) (182)

↔: correlation/association, ↑ higher infiltration/higher density/increase, ↓ lower infiltration/lower density/decrease. IHC, immunohistochemistry; mDCs, mature DCs; iDCs, immature DCs.

Several studies have investigated the prognostic value and distribution pattern of TIDCs in CRC patients’ tissues. DCs have been linked to both positive and negative effects on CRC prognosis, depending on their maturation status, location, and interaction with other immune tumor-infiltrating cells. Several studies have correlated a higher number of TIDCs with increased patient survival, lymphocyte infiltration, lower metastasis, and overall better prognosis (66, 149–154). In some of these studies, the S100 marker alone was used to identify DCs. These findings may be somewhat limited since S100 expression is restricted to only a few DC subsets, and is not a DC-specific marker being also expressed by other cell types including macrophages (183). Moreover, in these studies the maturation state of DCs was not assessed, which precludes information on DC pro- or anti-tumorigenic polarization and functions.

Further studies have investigated TIDCs distribution in CRC in correlation with their maturation status. Two studies report that the density of tumor-infiltrating mature DCs (mDCs) is lower in metastatic sites than in primary sites, which in turn is lower than in normal mucosa (158, 167). In addition, different studies have shown that mDCs are usually present in the invasive margin and cluster with T cells in lymphoid structures (TLS), whereas immature DCs (iDCs) are often more scattered through the tumor stoma (159, 160). These results suggest differential immune landscapes in primary and metastatic tumor sites and overall mDC exclusion from tumor sites.

Additionally, the maturation status of TIDCs has been correlated with disease progression and patient prognosis. Lower levels of mDC infiltrates have been linked to more advanced disease stage, higher metastatic burden, Treg infiltration, and poor prognosis. Conversely, higher levels of mDCs relative to iDCs, have been associated with stronger Th and CTL responses and better prognosis in general (19, 56, 165, 166, 168–171). These results are in agreement with the anti-tumorigenic potential of mDCs and the tolerogenic role of iDCs. In line with this, MSI tumors with better survival are characterized by an increase in mDCs and lower numbers of Tregs in comparison with MSS (163, 164). Two studies have shown somewhat contradicting results, implying a correlation between increased mDCs infiltration, and shorter survival and increased metastasis (172, 173). More recently, PD-L1+ DCs were clearly associated with CD8+ T cell infiltration and good survival in CRC (174). Interestingly, several studies have linked the observed defects of DCs and poorer survival with increased expression of COX-2, HMGB1, IL-6, and TGF-β by CRC (56, 66, 153, 161, 162, 167). Of note, a limitation of many of these studies is the use of a small set, and often non-DC specific markers to characterize DCs and their maturation status. Notwithstanding, these studies certainly provide valuable insight on TIDCs distribution and prognostic value in CRC patients.

Besides TIDCs, numerical and functional defects of circulating DCs in CRC patients have also been observed. In general and in relation to disease progression, a decreased number of circulating DCs, increased number of progenitors, and a higher iDC/mDC ratio in CRC patients have been reported (175–179). These defects have been associated with increased serum levels of TGF-β and VEGF (175, 176, 179). Furthermore, functional defects have been noted, including defective *ex vivo* differentiation and maturation of DCs from monocytes, tolerogenic phenotypes with decreased IL-12 and TNF-α release, increased release of IL-10 and TGF-β, and a compromised ability to induce allogenic T cell proliferation (179–182). These findings highlight the importance of systemic immunosuppression exerted by the CRC.

In addition, several studies have concluded that CRC explant tissue-conditioned medium inhibits LPS-induced *in vitro* DC maturation and function. In these assays, upregulation of co-stimulatory markers (CD80 and CD86) and PD-L1, and secretion of IL-12 and TNF-α was inhibited, while secretion of IL-10 was potentiated suggesting DCs acquire a tolerogenic phenotype (184–187). One study even correlated stronger inhibition of DC maturation by CRC-conditioned medium with poorer survival in patients (186). A variety of tumor-derived factors secreted by CRC including VEGF, CCL2, CXCL1, and CXCL5, were shown to mediate these effects synergistically (184, 185). Collectively, these studies demonstrate that CRC, mainly through soluble mediators, evades anti-tumor responses by exerting local and systemic immunosuppression and disabling both infiltrating and circulating DCs.

Strikingly, very few studies have focused on the different DC subsets, which present different functional specializations, in relation to CRC and to T cell function. For instance, infiltration by pDCs has yielded controversial results, with some studies associating pDCs infiltration with Treg development and poorer prognosis and others with increased survival, but without taking maturation status into account (155–157).

All in all, the reported findings illustrate the importance of local and systemic modulation of DCs in CRC. Currently, there are insufficient available data to elucidate on the complex mechanisms underlying DC dysfunction in CRC patients. To develop a more comprehensive picture of the implications of DC dysfunction in CRC, additional studies will be needed to determine the differential roles of the DC subsets in CRC, taking into account their functional specialization, maturation status and plasticity, which can have contrary impacts on tumor progression and prognosis (73). Moreover, further insight on the differences between metastatic and primary tumor sites will be of value.

## Tumor-Induced DC Dysfunction and Immunotherapy Efficacy

As illustrated in the previous section, the CRC immunosuppressive TME shifts the delicate balance of DCs from inflammation to tolerance, fueling disease progression and spread. In addition, DC dysregulation has also been implicated in patients’ unresponsiveness to immunotherapies, further contributing to a poor prognosis. Current immunotherapeutic approaches for CRC have been mainly focused on targeting T cells, either by immune checkpoint inhibitors or by stimulating T cell activating receptors (188). However, only CRC patients harboring tumors with high mutational burden - MSI, accounting for less than 5% of the patients with mCRC - benefit from these treatments (35, 36).

Interestingly, in MSS CRC patients tumor-infiltrating neoantigen-specific T cells have been detected despite their low mutational burden and low responsiveness to immune checkpoint inhibitors (189). This and other data suggest that, upon treatment, tumor-specific T cells can be generated but are not functional (47, 189, 190). This low T cell reactivity has been linked to a TGF-β-rich TME (189). Importantly, this T cell-centric approach of immunotherapy does not account for DC impairments, despite their crucial role in T cell priming, activation, and recruitment in the tumor bed.

Indeed, several studies have emphasized a strong dependency of effective immune checkpoint inhibition on correctly functioning and activated TIDCs. These studies confirm that cross-priming, licensing, and recruitment of T cells by functional intratumoral DCs is required for successful responses to anti-PD-1 therapy and T cell adoptive therapy. And, that this is mainly mediated by CXCL9/10, IL-12, and IFN-γ secretion by DCs (85, 123, 191–195).

In line with this, one study in a melanoma model has shown that expansion and activation of TIDCs at the tumor site by recruiting and activating agents such as FLT3L and poly I:C, enhanced therapeutic response to immune checkpoint inhibitors (196). In addition, a recent study indicates that blocking CRC-induced WNT2 secretion by CAFs restores DC functions enhancing anti-PD-1 efficacy (197). These studies emphasize the importance of functional DCs in effective intra-tumoral DC-T cell crosstalk for immunotherapy response. Therefore, targeting T cells without taking into account and resolving DC dysfunction might hamper the success of T cell-centered immunotherapies in CRC (198, 199).

Additionally, other studies show that NK cell and DC reciprocal interactions are required for enhanced T cell responses and determine responsiveness to immune checkpoint inhibition treatment (200, 201). NK cell frequency correlates with enhanced DC infiltration in the tumor, which in turn correlates with patient responsiveness to immune checkpoint inhibitors and increased survival. Moreover, studies have shown that DC vaccine efficacy is strongly dependent on NK cell activity and DC-NK cell crosstalk (202–204). This highlights the importance of NK cells in tumor immunity not only by their ability to kill cancer cells directly, but also as promoters of DC activity.

In the clinic, studies with DC vaccines and other DC-targeting therapies in CRC have yielded modest results. DC vaccines consisting of *ex vivo* activated patient DCs, tailored against tumor-associated antigens, have the potential to trigger and boost T cell anti-tumor responses. This highly specific approach, combined with a relatively low risk of toxicity, makes DC vaccines particularly promising (10, 205). However, despite initial encouraging results including increased T cell responses and a good safety profile, DC vaccines have not shown strong therapeutic benefit in CRC patients (206–216). This is hypothesized to be linked to the strongly suppressive TME, particularly a TGF-β rich TME (10, 217, 218) and, consequently, defective and immunosuppressive DC populations. Besides suppressing administered DCs, these dysfunctional DC subsets can also limit T cell infiltration and effector function (96, 219). Current studies focus on improving vaccine platforms, increasing and expanding tumor specificity of vaccines, counteracting the host immunosuppressive mechanisms of resistance, and testing combinatorial therapies (220). Indeed, three ongoing or recently completed trials for mCRC aim to synergize DC vaccines with IL-2 (NCT02919644) or with immunological checkpoint inhibitors (NCT04912765 and NCT03152565). In conclusion, it seems that efficacy of immunotherapies is reliant on functional DCs for proper T cell-DC-NK cell crosstalk, which is disrupted by the strongly immunosuppressive TME.

## Reverting DC Dysfunction to Unleash Anti-Tumor Immunity and Response to Immunotherapy

Mounting evidence highlights the central role of DCs in anti-tumor immunity and consequently on immunotherapeutic responsiveness and urges the development of DC- and TME-targeted therapies to tackle DC dysfunction in treatment-resistant CRC patients. Unleashing DCs emerges as a crucial step to make immune checkpoint inhibition and other immunotherapies available to all CRC patients. The development of combinatorial therapies for mCRC is being actively sought since monotherapies have not demonstrated effectiveness in improving patient’s outcome (10). Since tumors evolved multiple mechanisms to avoid immune evasion, a multi-faceted approach focusing on different mechanisms will most likely be needed to address current issues in CRC treatment (10, 199, 221–223).

To breach the strong immunosuppressive CRC microenvironment, a promising therapeutic venue seems to include combinatorial strategies to in concert: 1) foster DC activation and function, boosting antigen presentation or TIDC abundance, 2) stimulate and unleash NK or T cells by immune checkpoint inhibition, and 3) target tumor-mediated mechanisms and tumor-released immunosuppressive factors. This should in principle allow overcoming of the strong suppressive TME, trigger more efficient NK-DC-T cell crosstalk and lead to full unleashing of local and systemic anti-tumor immune responses. As a metaphor this approach can be described as not only releasing the brakes of the immune system by lifting the vail of immunosuppression but also pressing the gas pedal by stimulating the key coordinators and effectors of immune responses.

The main aim of DC-targeting strategies is to skew the TIDC phenotype from tolerogenic to inflammatory, and enhance DC-intrinsic abilities. Strategies that aim to restore and stimulate DC functions, although not powerful in the clinic alone, might have a key role in combinatorial treatments (10, 224–228). Approaches to circumvent tumor-mediated DC dysfunction can consist of DC vaccines or directly targeting and stimulating DCs *in situ*, by delivering DC-recruiting or promoting agents such as FLT3L, CpG, TLR and STING agonists (199). In addition, reverting DC dysfunction would break the positive feedback loop of immunosuppression, allowing wider reprogramming of the TME.

Thus far, overcoming the TME remains the most important and daunting challenge for CRC. Despite promising leads on the apparent key role of tumor-released suppressive factors such as TGF-β, VEGF, and PGE2, it is still largely unclear how CRC shapes DC fate. A more complete understanding of the complex web of interactions and elucidation on key mechanisms in play between CRC and DCs is required. Future studies will certainly provide new rationales and open doors for the design of novel therapies to unlock the full anti-tumor potential of DCs, while sensitizing previously unresponsive patients to immunotherapy.

## Future Perspectives

There is abundant room for further progress in understanding CRC-DC interactions. In future studies, it is important to consider both the functional and phenotypical plasticity of DCs subsets, and the heterogeneity and complexity of CRC. It is well-established that different DC subsets can have distinct, either complementary or opposing, functions in anti-tumor immunity and hence affect tumor progression differently (73). To better dissect this heterogeneity, further research is required to characterize the functional status, quantify, and assess the distribution of the different DC populations present in the tumor sites and in circulation in CRC patients. Whether these are correlated to CRC molecular subtype, disease progression, prognosis, treatment response or immunosuppressive systemic factors needs to be determined. Possibly, different subsets have different predictive potential and might arise as novel biomarkers for disease progression and treatment response. To get a comprehensive overview, patient material from different disease stages and molecular subtypes, including tissue sections, fresh biopsies and blood samples, will be valuable.

Furthermore, future research should aim to explore and study both metastatic and primary sites. There are few studies with a comparative perspective lens between primary and metastatic CRC. It is important to address differences in DCs infiltration, phenotype and functionality, and tumor-mediated evasion mechanisms. Metastatic sites are often different not only at a molecular level but also in the immune landscape and the TME. Moreover, it is important to research metastases since these are often more resistant to therapy than their primary counterparts. As such, investigating and tackling the immunosuppressive environment of not only the primary tumor, but also the metastatic sites is of outermost importance (229).

At the molecular level, it is also important to gain insight on how CRC shapes the different DC subsets, what are the underlying mechanisms, and what are the local mediators of DC dysfunction. Also, many questions remain unanswered regarding how the different subsets correlate with each other and with T and NK cell effector function in CRC. To study in more detail these CRC-specific underlying molecular mechanisms, *in vitro* models are most suitable. Currently, there is a shortage of relevant and representative *in vitro* models to study the CRC TME.

Recently, patient-derived organoids (PDOs) are emerging as a powerful tool to study CRC heterogeneity and therapy responses by faithfully recapitulating many of the traits of patients’ disease. Moreover, studying DCs in a tumor organoid context is still a largely unexplored field with a lot of opportunities. As such, 3D co-culture systems of DCs and CRC PDOs seem a promising approach to more closely study their interactions. In order to more representatively model the biological context and the *in vivo* interactions, higher complexity 3D co-culture models including stroma cells and different immune infiltrates that mimic the complex structure and composition of a tumor and its microenvironment are sought after (104, 230–232). In this line of research, recently a complex organotypic skin model was successfully developed to study DCs in melanoma (104). Hopefully in the near future, similar organotypic or complex 3D organoid based co-culture systems can be developed to study the CRC TME, both primary and metastatic, and follow the behavior of different subsets of DCs.

In addition, these models can possibly achieve sufficient physiological relevance to serve as testing platforms for novel therapies. This would be valuable since there are many unexplored combinatorial opportunities including TME- and DC-targeting therapies for CRC. It also remains to be determined which DC targeting strategies are effective and synergize with other immunotherapies in CRC patients. Furthermore, co-culture systems with PDOs offer the opportunity to test and tailor combinatorial strategies in a patient-specific manner.

## Conclusions

Metastatic CRC remains one of the most aggressive and lethal cancers, with the large majority of patients being refractory to therapy. Disease aggressiveness and resistance to therapy has been linked to the tumor genetic makeup and a highly immunosuppressive TME. DCs have key roles in anti-tumor immunity, making them crucial targets for tumor evasion mechanisms. Overall, literature suggests that CRC-induced DC dysfunction is decisive for: impairing anti-tumor immune responses, tumor progression, metastatic colonization and initiation, and unresponsiveness to immunotherapies such as immune checkpoint blockage.

Unravelling the complex crosstalk between CRC and DCs and determining its significance for patients holds promise for identifying and modulating key mechanisms involved in disease progression. This opens doors for the design of novel strategies to reverse DC dysfunction. In principle, restoring DC functions can unlock the full anti-tumor potential of DCs and hence, unleash systemic anti-tumor immunity mediated by T and NK cells against primary and metastatic CRC. This approach should make immunotherapies available for more patients. Therefore, reverting DC dysfunction emerges as a promising path for CRC treatment and a critical pillar for combinatorial strategies. In order to design novel therapies, a completer and more comprehensive overview of the CRC TME and the mechanisms driving tumor progression and induction of DC tolerizing properties is necessary. For futures studies, examining patients’ tissues and blood and development of *in vitro* TME co-culture models based on PDOs appear as promising tools to obtain the missing knowledge.

## Author Contributions

BS performed literature searches and wrote the first version. All authors contributed to the article and approved the submitted version.

## Funding

DT is funded by a Hypatia Tenure Track Fellowship grant from the Radboudumc and by the Netherlands Organisation for Scientific Research (NWO/ZonMW VIDI grant number 91719371). IV is funded by EU grant Oncobiome (825410) and Health Holland/SGF grant DC4Balance (LSHM18056-SGF).

## Conflict of Interest

The authors declare that the research was conducted in the absence of any commercial or financial relationships that could be construed as a potential conflict of interest.

## Publisher’s Note

All claims expressed in this article are solely those of the authors and do not necessarily represent those of their affiliated organizations, or those of the publisher, the editors and the reviewers. Any product that may be evaluated in this article, or claim that may be made by its manufacturer, is not guaranteed or endorsed by the publisher.
